# Influential Factors when Making Decisions About Dementia Medications in Memory Assessment Services; a Focused Ethnography and Interview Study

**DOI:** 10.1177/08919887251362465

**Published:** 2025-08-02

**Authors:** Rachael Kelley, Claire A. Surr, Gregor Russell, George Crowther, Rebecca Dickinson, Jemima Dooley, Alys W. Griffiths, Peter Knapp, Sarah J. Smith

**Affiliations:** 1 Centre for Dementia Research, 4467 Leeds Beckett University, Leeds, UK; 2 Consultant Old Age Psychiatry, 1910 Bradford District Care Trust, Bradford, UK; 3 Consultant Old Age Psychiatry, 4471 Leeds and York Partnership Foundation Trust, Leeds, UK; 4 4468 The University of Leeds, Leeds, UK; 5 Mood Disorders Centre, University of Exeter, Exeter, UK; 6 Sheffield Institute for Translational Neuroscience (SITraN), 7315 University of Sheffield, Sheffield, UK; 7 Hull York Medical School & the Department of Health Sciences, University of York, York, UK

**Keywords:** dementia, pharmacological treatment, shared decision making, memory assessment

## Abstract

**Background:**

Discussing pharmaceutical treatment for dementia is challenging because of variation in disease progression, lack of curative treatments, and communication difficulties. Research in the context of dementia suggests shared decision making is limited, this study examined how dementia medications are discussed in practice.

**Methods:**

Focused video/audio ethnography of clinical appointments (n = 14), semi-structured interviews with patients/supporters (n = 23) and clinicians (n = 5) were employed to examine communication practices.

**Results:**

Two themes developed; *Framing and understanding of information in the context of uncertainty* explores how uncertainties around risks and benefits are understood. *‘Not worth the risk’* or ‘*nothing to lose*’ presents how patients/supporters and clinicians balance individuals’ contexts/perceived risks/benefits. In the absence of certainty around potential benefits, risk often informed decision-making, particularly for frailer or more vulnerable patients.

**Conclusions:**

Clinicians should be aware of their influence on decision-making and be cognisant of the way that they frame opinions, which are largely based on clinical experience. Prescribers would benefit from a standardised information source which enables them to describe the likelihood and magnitude of benefits and side effects in a universal way. Accessible information for patients and relatives about the same is also recommended. Patients and relatives make their decisions to take medications in the context of relative uncertainty about the likelihood of benefits, with risk playing a pivotal role in decision making for some.

## Background

In the UK, people with suspected dementia are typically referred to Memory Assessment Services (MAS). Following an eligible dementia diagnosis (typically Alzheimer’s type dementia or mixed dementia) MAS will, where appropriate, offer treatments for dementia. As of 2025 in the UK, these take the form of 4 approved medications: donepezil, rivastigmine, galantamine (cholinesterase inhibitors; AChIEs), and memantine.^1^ Pharmacological treatment for dementia is challenging as the treatments are not curative and it is difficult to predict who will benefit because of individual variation in progression of the condition. Donepezil, the first line treatment, has been shown in clinical trials (and practice) to have limited side effects, and has modest benefits in terms of symptom management.^2^ Recently a new generation of disease modifying dementia treatments have become available, Lecanemab (a human monoclonal antibody drug) has been licensed for use in the UK by the Medicines and Healthcare products Regulatory Agency (MHRA) but has not been recommended for use in the NHS at this time due to the benefits being judged to be too small to justify the costs of administration and monitoring of side-effects^3^ - like older medicines, Lecanemab has modest benefits, will only be suitable for a small number of people with dementia and can have side-effects that require regular monitoring. For example, side effects of this medication can include swelling or bleeding in the brain that are usually mild to moderate but can sometimes be serious.^4^ Lecanemab is likely to be the first of a number of new pharmacological treatments for dementia set to become available over the coming decades, each of which may have different benefit and risk profiles which will require consideration in decisions to prescribe and take such medications.

Clinicians in the UK have a legal obligation to explain the risk of harm and likelihood of benefit of treatments to patients to support informed decision making.^5^ Discussing and making decisions about whether to initiate pharmaceutical treatment for dementia can be complicated by the variation in disease progression, lack of curative treatments^6^ and the cognitive and communication difficulties dementia can bring. Improving risk and benefit communication to patients has been an increasing focus of regulatory agencies and scientific organisations over the last decade^7,8^ to better support informed shared decision-making. Shared decision making incorporates 3 elements: recognising and acknowledging that a decision is required; knowing and understanding the best available evidence (including the risk and benefits); and incorporating the patient’s values and preferences into the decision.^9^ It has elsewhere been described as ‘an approach where clinicians and patients share the best available evidence when faced with the task of making decisions, and where patients are supported to consider options, to achieve informed preferences”^10^. Shared decision-making (SDM) is mandated in the UK,^11,12^ and requires that treatment initiation is collaborative; based on evidence-based medicine, clinical expertise and what matters most to patients.^11^ SDM has the benefit of raising compliance with agreed treatment, improving patient safety, reducing health inequalities and curbing overprescribing.^13^

There are few studies examining the involvement of people living with dementia in decision making about initiating treatment for dementia. One study directly examined shared decision making within MAS at the point of prescribing – finding that the way in which medications are recommended by doctors did not affect the patient’s reaction in terms of accepting or resisting the proposed medication; most patients (80%) actively or passively resisted the recommendation to take medication. Furthermore, there was no association between patient acceptance or resistance and whether medication was prescribed; medication was just as often prescribed when patients resisted as when they accepted. Patient satisfaction was found to be reduced when patients were offered medications without a clear choice or invitation. Overall, evidence of opportunities for shared decision making in this study was limited.^14^

Shared decision-making with people with dementia can be less straightforward than with the wider population. Their involvement may be compromised by the complexities in describing the outcomes of pharmaceutical treatment. For example, to reduce potential distress that explanations may cause, clinicians may employ euphemistic language,^15^ that in turn can be difficult for people living with dementia to understand.^16^ A further complexity is the role family members may need to have in the decision-making process and how to ensure effective triadic communication between the clinician, patient and relative.^17^ There is evidence of addressing such complexities thereby enhancing shared decision making for people living with dementia through the use of patient decision tools in the context of deprescribing.^18^ Ailiabouni and colleagues showed that a co-produced consult patient decision aid could help people living with dementia review their goals and engage in a shared decision making process around the continuation or deprescribing of Cholinesterase inhibitors

Notwithstanding the challenges, the goal should always be to facilitate the involvement of people in their decision to take medications recognising their beliefs and preferences. This qualitative study was conducted as part of a wider study into how pharmaceutical treatment for dementia is communicated in MAS. It sought to explore how dementia medications are discussed in MAS clinic appointments, focusing on the factors that influence decision making around taking dementia medications and to make recommendations that could help to improve the shared decision-making process.

### Patient and Public Involvement

A lived experience advisory group was established consisting of 6 people with personal experience of attending MAS for a dementia diagnosis; either their own diagnosis (n = 3) or the diagnosis of a relative (n = 3), residing across England. The group met 6 times during the project, via videoconferencing. Members contributed to and influenced the design, delivery and analysis of the study, for example by advising on content of interview topic guides, discussing recruitment and consent processes, reading and commenting on data excerpts to support analysis and contributing to dissemination plans and outputs.

## Methods

This study was qualitative and used focused video ethnography (see^19^ for use in health research) utilising transcripts of video and audio observations of clinical appointments in MAS, and semi-structured interviews with patients, their supporters and clinicians. The services were located in 2 NHS Trusts in the North of England, serving inner city and rural areas, including areas with ethnically diverse populations.

### Sampling

The sample used in this study is taken from a larger sample of n = 29 appointment observations; we only focused on observations that had accompanying interviews with patients who attended the appointments for this paper (n = 14).

### Participants

Fourteen patients who received a dementia diagnosis and 16 relatives/supporters participated, contributing 14 clinical encounters and 23 interviews. Their demographic details are provided in Table 1. Eight MAS clinicians (psychiatrists of various grades) provided 1 or more of these appointments and 5 took part in interviews.

**Table 1.** Patient and Relative/Supporter Demographics.

	Patients	Relatives/Supporters
	N (%)	N (%)
Diagnosis		
Alzheimer’s disease	11 (79%)	
Mixed dementia	3 (21%)	
Age	*	**
30-39	-	2 (13%)
40-49	-	1 (6%)
50-59	-	3 (19%)
60-69	1 (7%)	4 (25%)
70-79	6 (43%)	1 (6%)
80-89	4 (29%)	1 (6%)
Gender		
Female	11 (79%)	12 (75%)
Male	3 (21%)	4 (25%)
Ethnicity	***	****
White British	8 (57%	8 (50%)
White Welsh	1 (7%)	1 (6%)
White Scottish	-	1 (6%)
White New Zealand	1 (7%)	-
Black Caribbean	1 (7%)	1 (6%)
Mixed Heritage	-	1 (6%)
Relationship		
Partner/Spouse		5 (31%)
Daughter		8 (50%)
Son		1 (6%)
Granddaughter		2 (13%)
**Total**	**14 (100%)**	**16 (100%)**

Missing data: *n = 3 (21%); **n = 4 (25%); ***n = 3= (22%); ****n = 4 (26%).

Staff participants were predominantly Consultant Psychiatrists (n = 6) or staff grade/specialty doctors (n = 2). They had worked in their MAS roles for between 10 months and 14 years, and their total experience of working in MAS services ranged from 1.5 to 14 years.

### Observations

As data collection occurred during the COVID-19 pandemic a range of in person and remote methods were employed for the observations. MAS appointments took place remotely using video software (n = 4) or telephone (n = 1), and in person in clinic settings located within NHS sites (n = 8) or during home visits (n = 1). Appointments involved clinicians, the patient, and their relative/supporter(s). Appointments were audio or video recorded either via the video-calling software used for the appointment, a separate video-recording device set up in the same room, or via a dictaphone to record audio only, dependent on participant preference, practicability and COVID-19 restrictions at the time. Researchers were not present in the room during any of the appointments; video recording equipment was set up and started by the researcher ahead of the appointment, with clinicians responsible for operating Dictaphones and recordings undertaken via video-calling software.

### Interviews

All participants who took part in an appointment recording were invited to participate in up to 2 interviews. Interviews were conducted by 2 of the authors RK, AWG with the same researcher conducting both interviews where possible. Researchers conducting interviews were employed by the University and were not involved in patient care and so had no professional relationships with any of the participants (patients, families or clinicians). Interview 1 explored the information about medications discussed in the appointment and their decision-making about whether to take the medication. Interview 2 explored experiences since and views on this decision. Interviews were conducted with the patient and carer(s) as a dyad/group or with just carer/supporter(s) if the patient was unable to participate. They were held in-person in the patient/carers home or other location of their choice, or by video conference or telephone, dependent on participant preference and COVID-19 restrictions at the time. Interviews with clinicians were held at the end of the study to explore their views on discussing and decision-making about dementia medications with patients and their carer/supporter(s).

### Data Analysis

Given the focus on information provided during appointments, perceptions of this and how it informed decision-making, only data from participants who had an appointment transcript and at least 1 interview were included in the analysis. The video and audio recordings were transcribed verbatim and pseudo-anonymised at the point of transcription. Appointment transcriptions included verbal utterances and notable non-verbal behaviours such as body language, gestures, eye direction. Only the section of the appointment in which medication discussions occurred were transcribed – all medication discussions referred to dementia medications - cholinesterase inhibitors and/or memantine. They were analysed using reflexive thematic analysis^20^ by RK and AWG who were employed by the University and are not clincians, with input at the familiarisation and code development stage from an independent researcher and members of the lay advisory group (see above). Anonymised quotations were presented at 2 of the meetings where all members were present. Participants discussed the meaning of quotations in the context of the developing themes and sub-themes, providing insights into interpretation and also potential discussion points to be raised.

Analysis began with a process of data familiarisation, where a subset of appointment and interview transcripts were read and discussed in-depth by [RK & AWG] to develop initial analytic ideas. These ideas were then developed further by repeating this process with another subset of appointment and interview transcripts. Following this, a further meeting took place to develop and refine an initial coding framework. This was then used to code all transcripts using NVIVO software. The coding framework, and additional codes, were developed, discussed and refined, as required, and analysis notes made on the developing codes. Following the coding process, further meetings were held also including RK, AWG to develop and refine themes and sub-themes from the coded data, and later to review and refine draft write-ups of the themes and subthemes.

### Ethical Issues

Ethical approval for the study was gained from the National Research Ethics Service [panel details RK, AWG] on 8.3.21. All participants gave fully informed written consent prior to data collection. Where participants had dementia an assessment of capacity to give informed consent was conducted in line with the Mental Capacity Act 2005. Where participants lacked capacity, advice about their wishes was provided by a personal consultee. Continued willingness to participate was checked at each subsequent data collection point along with mental capacity.

## Findings

**Figure 1.**
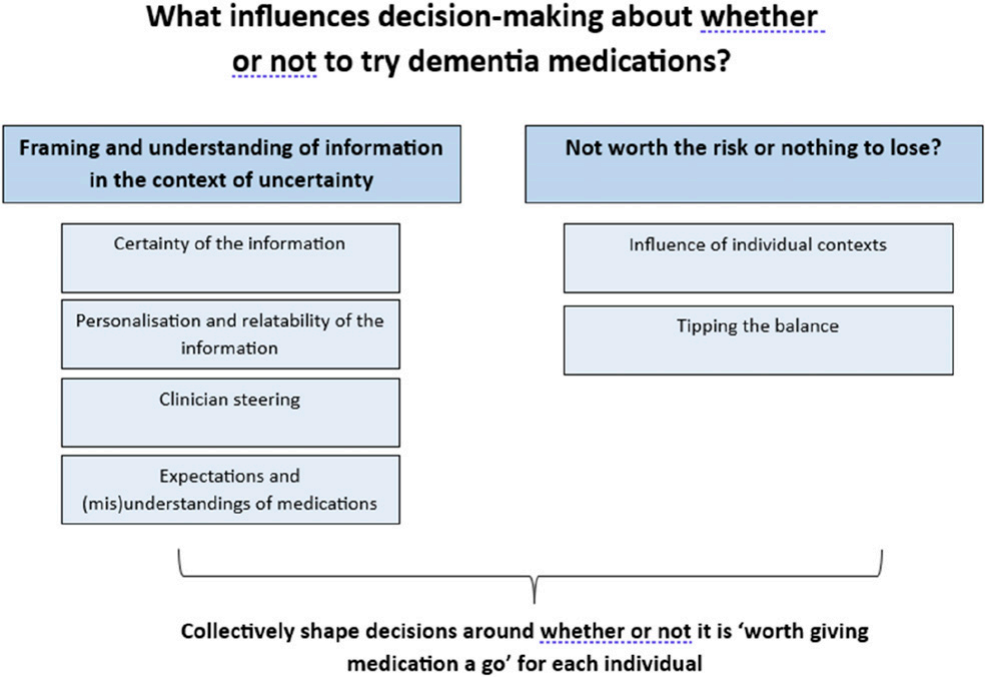
Organising themes.

Two organising themes were developed, in accordance with the analysis plan, to explain how presentation of medicines information informs decision-making. The first theme discusses how medicines information and the uncertainties around its risks and benefits is presented and understood, and how this informs decision-making: ‘*Framing and understanding of information in the context of uncertainty’*. The second theme explores how patients, supporters and clinicians balanced individuals’ contexts and the perceived risks and benefits to the individual, to inform decisions on whether medication was: *‘Not worth the risk’* or that they had ‘*nothing to lose*’. In the absence of certainty around the potential benefits of current dementia medications, whether any potential risks to the individual from taking the medication were perceived as high or low (for example, their ability to tolerate and manage potential side effects) was a key influence on decision-making. Collectively, these organising themes fed into participants’ decisions about whether taking or prescribing medications was ‘worth giving medication a go’ (Figure 1).

### Organising Theme 1: Framing and Understanding Information in the Context of Uncertainty

The information about medications provided during consultations, and obtained from other sources (eg, the internet, relatives or friends, or information leaflets), was central to informing decision-making for people diagnosed with dementia and their supporters. We identified notable characteristics in how medicines information was presented in relation to sub-themes of the *‘Certainty of the information’*, the ‘*Personalisation and relatability of the information’* and the level of *‘Clinician steering’* towards a particular decision, all of which impacted upon how patients and their supporters understood and used the information to decide whether to take medication. The influence of ‘*Expectations and (mis)understandings of medications’,* gained both from clinicians and from other sources, were also influential in the decision-making process.

#### Subtheme 1: Certainty of Information

Uncertainty could be present at multiple levels, for example in relation to presenting the nature, likelihood and degree of benefits or side effects of taking the medication; conflicting information provided in appointments vs that sourced elsewhere; and the extent to which people with dementia and their family members were able to understand and process the information provided. Medication was often discussed immediately after a diagnosis was given, or alongside cognitive, hearing or other communication difficulties which could compromise people’s abilities to process the required information.

The uncertainty associated with taking dementia medications is inherent, given it is hard to predict who may benefit from taking medication and how much. One of the clinician’s roles was to try and communicate this uncertainty – which was evident in many of the consultations we observed:

C: Yeah? So, it doesn’t cure the underlying problem but it’s just treating some of the symptoms of the brain. So, a lot of people may not see any change in the way they are. Some people might see some improvement, very little. And some people are unable to tolerate the drug to see any effects.

In this consultation the relative shares an understanding from the initial information, which the clinician identifies as inaccurate and has to address with further explanation.

R: Does it? So, is it really trying slowing things down a little bit, slowing the progression?

C: It doesn’t, actually in real terms, it doesn’t slow things down but its, it, the, it maintains the function so…

... the symptoms which you have currently, you may have less, you may have the same. (B101& B201, Clinician, Person with Dementia and Relative; Consultation)

Patient and family participants also, however, identified (and we observed) variability in the clarity with which treatment uncertainty was communicated, and in the level of detail provided regarding potential benefits:

R: I would suggest he didn’t really tell us much about what it would do other than a few side effects, if it would help. So yeah, so I felt that we didn’t know much about the medicine that was suggested

...and he did tell us a little bit about side effects

I (B109 & B209 Person with Dementia and Relative; Interview)

We also observed communication about benefits and risks without uncertainty communicated, such as definitive statements about outcomes. Given that inherently the medication outcomes are uncertain, such descriptions could be misleading or steer decision making (as discussed further in *’Clinician Steering’)*. For example:

We’re hoping to stop things getting worse but also you’ll always be a little bit better off taking the medication (Clinician A013)

Our data also contained examples of how medicines can be discussed in ways that can help to mitigate uncertainties, which could be exacerbated bycognitive impairment and potential distress associated with receiving a dementia diagnosis. For example, repeat appointments with the same clinician enabled relationship building and prior warning that a dementia diagnosis, and the offer of medication, was possible. This, when present, provided a staged approach to information giving and decision-making that people with dementia found beneficial:

‘…That last meeting with Dr X., was that when he kind of confirmed it, he did say on that previous letter that he thought it probably was [dementia], he thought that they thought that that's what it would be. So, it obviously wasn't a surprise to him [A012]’. (A012 & A013 Person with Dementia and Relative; Interview)

As opposed to the following experience:

R: I think when she said Alzheimer's I were a bit taken back, you know, So, I don't know if you know it, maybe a little bit more time could be given to... sort of absorb that information before you talk about the medication.

(A037 & A038 Person with Dementia and Relative; Interview)

Clinicians, people with dementia and supporters also identified other examples of information being communicated well and with little uncertainty, characterised by features such as clear and well-balanced information provided verbally and in written form, with opportunities to check understanding:

I thought it was quite straightforward. I didn't feel anybody was trying to baffle me with science or overload me with information…. and when he sort of drew it to a close, and then he said to dad, did he want to answer any questions, I thought, yeah, that's been very clear, quite concise, not too much information, but enough information…. (A012, Person with Dementia and Relative, Interview)

Where understandable information, either verbally or in writing, wasn’t provided, participants commented on how useful this would be:

‘It would be useful to have a leaflet or some information very, very specifically for the person with dementia rather than a carer or somebody wanting to find out information,’ (Interview carer A027)

As the above quotes collectively highlight, there appeared to be both necessary and unnecessary uncertainty present in, and surrounding, medication discussions. Approaches to reduce any unnecessary uncertainty (such as clarity wherever possible, a staged approach, opportunities to check understanding and providing written information to accompany that provided verbally), are possible and valued by families and people living with dementia.

#### Sub-theme 2: Personalisation and Relatability of the Information

To aid understanding clinicians spoke of trying to frame medicines information in ways that were relatable and tailored to the individual’s context, and this was also evident in appointment recordings. For example, clinicians could use explanations that were tailored towards their knowledge of the individual’s dementia symptoms or preferences regarding medication or level of detail:

C: ‘I think you do adapt your communication style to how your patient is... I suspect you are looking at someone and listening to someone and you will adapt how you explain it, to what you think probably may be more beneficial to them, or what you think they're thinking.’ (Staff Interview, A016)

Family members also sometimes aided the provision of information about the medication that was more understandable or relatable for their relative:

R: This medication you are talking about is similar to the 1 what Daddy’s taken for his Parkinson’s. (B118 Relative, Consultation)

As B045 also highlights, having trust and confidence in the clinician, and therefore in the information they provided, was also helpful in creating conversations that were relatable and easier for participants to understand:

‘I think she [Dr A016] can talk on a level that my mum can understand… I think if [Dr A016] hadn't spent the time she spent initially, I don't think that they'd ever have formed a relationship, and I think mum would have needed more and more appointments [to make a decision].’ (A045 Relative, Interview)

This supported participant’s confidence in decision-making about taking medications.

#### Sub-theme 3: Clinician Steering

Clinicians were sometimes noted to make potential steers in favour of taking the medication or not. We observed this ‘steering’ to happen in a variety of ways; consciously and unconsciously (as reflected on in intereviews). For example, by presenting the risks and benefits in a particular order,

‘...if you think it's going to be beneficial to the patient, I suspect that's why I do it first [talk about benefits], because I probably would prefer them to be on it. So I probably gear it that way. If I really think about it, it's probably what I'm doing. Because actually if it's not gonna be beneficial to someone, I will probably say the side effects of the medication outweigh the risks, and the probably say that first… So, yeah, I'm probably gearing it towards which way I prefer the patient to go, … but that doesn't mean the patient always agrees, or disagrees.’ (A016, Staff interview)

through positive framing (focusing on the benefits/minimising side effects),

And for some people actually it seems to put the condition in reverse for a bit, where memory actually improves noticeably to people. Things that you might have forgotten seem to be recalled. So that can happen too. (A013, Clinician, Consultation)

negative framing (focusing on not benefiting/side effects) or speaking in ways that made it clear they assumed the person would want to try the medication:

[Patient has just been told their diagnosis of AD by clinician]

C: So there are a few treatments we can look at. So certainly there is no cure for dementia yet, but the good thing is there are medications which can help.’ (B026’s Consultation)

The impacts of these steers on decision-making could be seen in appointments and interviews. For example, earlier in B118s appointment (quoted below), B118 and their family initially indicated being unlikely to take the medication, but following presentation of the side effects as minimal, short-lived, and manageable, they changed their decision.

C: ‘…not everybody benefits from these but like 2/3 of the people report a benefit ...

C: Erm, and side effects generally wear off after a few days of starting them

R: Okay

C: So that’s again an advantage

R: Yes, once the body gets used to them

C: Exactly, erm, before I give a script of, would you like to go on this medication?

R: I think it’s worth a try (B118’s Conulstation, Clinician, Patient & Relative)

In contrast, in B101s appointment below the medication was positioned less positively, with the likelihood of benefit portrayed as minimal against a stronger likelihood of risks, with B101 deciding not to take the medication:

R: We’re just saying that there’s a, there’s a medication here that you might be able to take. To help some of your symptoms with your memory.

P: Oh yeah.

R: But there are some side effects to it.

P: Oh right.

R: Such as you can get bad headache, or you can feel nauseous, or you might get an upset tummy so you have diarrhoea. Or, you know, loss of appetite, you might not feel hungry, or dizzy.

P: So I’d be better off not taking it do you think?

R: Well, it depends. You could, if we decide it would be a good idea you’d start with a very small dose and see if that’s ok, without any side effects. Because it can also have an effect on your heart, it can slow your heart rate down. So really, it’s a delicate thing to do, to work out whether it would be a good thing to try because some of the side effects might be severe and if the side effects are worse than the benefits that you’re getting then it probably wouldn’t be a good idea for you take it…’ (A101’s Consultation)

In contrast, there were also examples of more balanced presentations of risks and benefits that encouraged people to ask further questions and make decisions in either direction:

C: ‘One of the considerations is whether we use tablets to help with the memory... I think it’s also about recognising that... we need to make sure it’s having a benefit and not causing any problems. …. I say this because I’m very mindful of your bowels and what you were telling me before.…. I can give you some information for you to have a think about it… or, if you think this is something you’d like to do, then I’m more than happy to ask the GP to start it from today.’ (A037’s Consultation)

Carers/supporters discussed how trust in the clinician could accentuate the impact of steers towards the risks or benefits of medications to further influence decision-making about whether to commence medication:

The doctor knows better than I do about how this may affect my mum because she, you know, she sees lots of patients, she will probably be aware of people who have taken it and what’s happened to them. I don't know that so I have to take a lot of things on trust, which I do (Interview B221 carer)

This could assist in providing reassurance where there was uncertainty as above, or provide an authoritative steer towards a decision as below.

my dad’s very compliant most of the time, but my mother-in-law definitely wasn’t. And so, that’s what we wanted was somebody to tell her that, … you need to do this because she wouldn’t accept it from us, …. So, I do think a professional recommending it, I have no problem with that. (Interview B027 carer)

These examples highlight the influence that ‘*clinician steering’* can have on decision-making, and, therefore, the importance of clinicians being aware of the potential influence of the steers they may consciously or unconsciously deliver, for example, via information ordering, assumptions, or emphasis on risks or benefits, as these are often likely to be followed by patients and families.

#### Sub-theme 4: Expectations and (mis)understandings of Medications

Medicines information was interpreted by patients and families in a range of ways and with varying degrees of accuracy. For example, some patients and families arrived at appointments with pre-existing expectations or (mis)understandings about dementia medications, whilst others clearly misunderstood the information provided, all of which was dealt with variably by clinicians:

P: Oh, is that the one [medication] that makes you aggressive?

R: Well yes. That’s what I mean. When we looked it up, the first possible side effect was aggression.

P: And I was like, that’s the last thing I want to be. I’m not like that.

C: I’m surprised what Google comes out with. Um

P: You don’t know?

C: No! No. I can show you the leaflet. I mean very rarely some people can have hallucinations and things like that.

(B103 (Patient) and B203 (Relative), Consultation)

How misunderstanding such as this were dealt with had important impacts on decision making. For example, after their appointment, B103 (patient) and B203 (relative) felt medication probably was not for them due to their fears about B103 becoming aggressive; a concern that was unlikely to occur in reality but was not resolved by the clinician’s response:

P: ‘I think I’m leaning towards not taking it. Just because at the moment, managing my forgetfulness is easy enough and I just, ... didn’t like that aggressive bit….’

R: ‘I certainly don’t want you to take something that’s going to be aggressive, because I want to be with you as you are…‘

(L103 and L203 interview patient and carer)

Ultimately, B103 decided to take the medication following further reassurance from a memory nurse that aggression was a highly unlikely side effect.

Pre-held expectations in relation to medication were also possible, for example from prior experiences with medications for other conditions. Some participants spoke of presuming that an effective treatment would be available and offered to them, *R: I think grandad thought it’d make you better – I think he thought your memory would improve from taking them but they..*

P: But it hasn’t done has it?

R: No. Maybe that’s because I didn’t explain it very well to him.’ (Interview A021 (Patient) & A023 (Relative)

Whilst others came having already decided medication wasn’t for them or may have a limited impact:

R: ‘My thoughts were that … it's not going to be a cure all. It's gonna be a suck it and see thing anyway, because that's the way medicines often go.’ (A012 Relative Interview)

Partial- and complete misunderstandings of medicines information provided by clinicians during appointments could also occur, :

R: Obviously, we want to get on to the treatment side of it, that’s what we’re really interested in.

C: Yes so …, we do have some medications. The purpose of medication is, that this medication enhances the chemical messenger over there, yeah. And it helps in learning and memory, and overall functioning, you know. So, we have got 2 groups of medication, one is acetylcholinesterase inhibitors. … and another one is a receptor blocker. So, here I’m interested in the first group first because that’s what we use mainly. And, if it doesn’t work or if you've got any side-effects then we will go to second group. Is that ok?

With complex or technical explanations such as the above hampering understanding:

R: That’s ok.

C: Yeah. Sorry?

R: Is there an email or a letter, because I don’t understand.

(A029, Patient, & A030, Relative, Consultation)

In other cases dementia-related cognitive impairment impacted understanding and recall:

C: So, I can give you a prescription for 4 weeks, then after that you’re going to phone your own doctor.

P: And what’s that for then?

C: For your memory.

P: Memory?

R: Memory.

P: Oh.

(B109- Patient and B209- Relative, Consultation)

As these expectations and interpretations often helped to shape decision-making, the extent to which clinicians explored and clarified them (where necessary) had a notable impact on decision-making, which we saw demonstrated to varying degrees in practice:

C: Are you with me with the discussion around the tablets?

P: Er, [pause] well, I’m not up to tablets. I’m gonna have to take them I know. So, that’s something that I am concerned about, but.

C: But the tablets about the memory which we discussed just now. Did it make any sense to you?

P: The tablet itself? No, because I haven’t studied it yet.

C: Oh, so you’re going to look at it later?

R1: Yes, I’ll go over it again.

(A026-Patient A028 – Relative, Consultation)

Clinicians taking the time to check and discuss any concerns or questions to ensure information had been clearly and correctly interpreted was, therefore, an important part of identifying and responding to potential mis-understandings.

### Organising Theme 2: ‘Not Worth the Risk or Nothing to Lose?’

How patients, supporters and clinicians balanced perceived benefits and risks of taking the medication for an individual shaped decisions on whether trying medication was ‘not worth the risk’ or, where risks were felt to be low or absent, that there was ‘nothing to lose’. In reality this was a continuum, or weighing scale of risks and benefits, rather than a binary judgement. Understanding and weighing up the risks and benefits to the individual made the *‘influence of individual contexts’* key to making a decision. In the absence of certainty around the potentially modest benefits available medications, the presence of perceived risks to the individual was often a principle deciding factor, which could lead to ‘*tipping the balance*’ over towards a decision to not to take the medication, where this was felt to outweigh any potential benefits.

#### Sub-theme 1: Influence of Individual Contexts

Considerations at an individual patient level determined perceived levels of risk, and ultimately influenced decision-making on whether taking medication was ‘not worth the risk’ or worth trying because as A021 expressed *“I haven’t got anything to lose have I really”* . Perceived risk was important and meant that some patients did have ‘something to lose’ which influenced the steer clinicians offered, as summarised by A016:

‘I'm thinking of the patient in front of me, where they're living at this moment. Who's around them? How practical it is to have the medication, what their past medical history is… Are they going to get the side effects or not? …Because it might not be right for everybody. …if we know they have certain medical conditions, or if they're sometimes not great at taking tablets, or struggled with adverse effects, there’s a stronger possibility that tablets aren't going to be the thing for them. And you sometimes have to recognize it might not be a way forward.’ (A016 staff interview)

As A016 highlights, an individual’s health and co-morbidities, including frailty, could increase perceptions of risk given the potential impacts even temporary side effects such as gastrointestinal problems, nausea or loss of appetite could have on the person’s physical and emotional well-being. B201 described how potentially limited benefits, heightened levels of perceived risk or negative impacts of taking an additional medication, which was an important influence on decision-making:

‘…We’re quite happy with the way she [Mum] is now, she is going to be 89 in a couple of months, s... she’s getting frailer … so our plan it to just keep her as comfortable and as physically active as we can, , …if there was a sort of miraculously massive benefit to taking the medication, you know, that we would seriously consider it, but we just couldn't see, for my mum that it would be beneficial (B201 carer interview)

Whether a person with dementia, or their wider support network, was felt to be able to support safe and consistent medication taking was a key consideration in decision-making

‘I've got a lovely elderly gentleman. He's not taken any medication for years. Doesn't think he's unwell, and really well supported by his neighbours. I think medication would really benefit him... But .. the neighbours who are his lasting power of attorney were very honest with me and said, but he's not gonna take anything, and I said, no, I agree.’ (A016 staff interview)

As was an individual’s own ability or presence of support networks to monitor and manage potential side effects :

‘You'll see some people who are at risk of falls but are always with their partner. So even though you know that there is risk of falls, you may want to take that risk because it's mitigated.’ (B001 staff interview)

Thus the presence of support networks, could help to mitigate potential individual risks of trying medication, even where these were more serious potential risks such as falls. Clinicians also discussed other approaches to support medication and side-effects management including the use of patches to reduce side effects or alterations to the timing, dose or type of medication to support medication initiation or as alternatives when first line approaches were not successful:

‘When the problems [side effects] happen there’s quite a lot of options you can go down, … if the side effects can be managed by other means, by adjusting the time or by giving advice with regard to food habits... that's how we need to approach …there are quite a lot of options. (B009 staff interview)

This level of attention to considering, and mitigating for, where necessary, each person’s ability to safely take dementia medications and manage potential side effects indicated how influential these factors were in informing decision-making.

#### Sub-theme 2: Tipping the Balance

Collectively, the way medicines were discussed with people with dementia and their families including the degrees of uncertainty, expectations of what medicines might deliver, interpretations of information and clinician steering, alongside individual contextual factors that might impact potential risks and benefits, all fed into a balancing of overall risks and benefits to the individual, to inform decision-making. We found that perceived levels of risk were, for many participants, the primary influencing factor when deciding on whether or not to take or prescribe dementia medications. Where overall risks were perceived to be low, even when the benefits could also be limited, the perception was often that the patient had ‘nothing to lose’, thereby ‘tipping the balance’ towards taking the medicine. Higher levels of perceived risks however, often ‘tipped the balance’ towards deciding that medication was ‘not worth the risk’:

R: I guess it’s, difficult, first of all to gauge the benefits and obviously, it can help but it’s a difficult thing. If she starts getting headaches and feeling nauseous and she had diarrhoea everyday then it’d be counter-productive because she’d be feeling so physically uncomfortable that even, … if the medication was halting her deterioration a little bit. … feeling physically ill, you know it’s very important for her. …for instance she really enjoys her … food, so if she was feeling nauseous and she couldn’t eat and drink properly. ...C: Yeah, we’re looking at the quality of life at this stage ...

(B101 - Patient and B102- Relative, Consultation)

Thus the ‘knock on’ risks of any side effects were also an important consideration, meaning it was not just the side effects that were taken into account when making decisions, but also any resultant impacts for the person with dementia, or their family. These might include impacts on physical health, well-being, quality of life, or family carer burden:

between my son and my husband and me, you know, we actually managed to get it so he [Dad] wasn't having to take Imodium every day. Because apparently he's done that for years. ...then of course you get something else [a proposed dementia medication] that says… it might have gastrointestinal side effects, and we're thinking oh crumbs, not again’. (Interview A012 carer)

Although the potential risks, and their knock-on impacts, could be particularly influential in ‘*tipping the balance’*, the potential benefits of taking the medication were still an important factor in decision-making. Many participants clearly recognised the potentially limited benefits but were happy to try medication, providing it did not adversely affect the person:

P: Well, they tell me that everything has side effects and she said that I might not get any.

R: Probably won’t.

P: Probably not ‘cause she said it’s quite rare but you see, she says there’s no reason for me not to try it. I’ve got, she didn’t say you’ve got to try it, but I will try it because I haven’t got anything to lose have I really….

(A021- Patient & A023 – Relative, Consultation)

The potential for ‘knock-on’ benefits was also an important consideration that could tip the balance when weighing up whether to try medication. Notably, in the face of possible limited symptomatic benefits, a sense of hope and knowing they had done everything possible, was often cited as an important additional benefit medication could bring:

‘I suppose it's it does help me to feel like … we're doing everything that we possibly can,.. and I can accept that doesn't make everything OK, but it does sort of reintroduce a sense of control insofar as you can exercise it. .’ (A007 carer interview)

Thus medication could have psychological benefits for families as well as for the person with dementia, so even when a patient had experienced side-effects and their family felt their cognitive symptoms continued to worsen, remaining on medication could still be seen as the right option, because it still offered hope. A045s mum had experienced side effects of the medication, and they noted ongoing cognitive decline but still felt it was offering enough hope to be satisfied with their decision to commence it:

R: T...the reason why I think she's [Mum] happy taking it, she thinks herself it's doing us some good but it's obvious it's not really doing her any good... But it’s giving her hope... Which might be false hope, but it's giving her hope.’ (A045 carer interview 2)

Collectively, the findings suggest that perceived risks are highly influential in decision-making. When risks (including knock-on risks) to the individual are perceived to be low, then this tips the balance towards it being ‘worth giving medication a go’ in the hope of seeing some benefit, even in the absence of clear signs of this. If, however, there are sufficient levels of potential risk to cause a concern then this is likely to tip the balance so it is unlikely the medication will be considered worth trying, or worth continuing to be taken.

## Discussion

This study has identified that even for well-established and widely-prescribed medicines like acetylcholinesterase inhibitors and memantine, which have the potential to offer modest patient benefits with side effects often characterised by clinicians as mild and self-limiting, the decision-making around discussing, prescribing and taking such medicines remains challenging.

Our data describe how complex communicating the benefits and risks of medication in this setting is. The limited time that clinicians have within appointments also meant that for the clinicians the decision making was initiated before the appointment itself– taking a view on their opinion on whether to prescribe treatment or not - something the clinicians pointed to in the interviews.

Part 1 of this task is made more difficult as not only is the evidence base heterogeneous and prone to misinterpretation,^21^ but also that the objective benefits described in the literature (eg, objective improvement on cognitive tests such as MMSE), mean very little to patients – and extrapolating this into meaningful information relies on clinicians judgement and is inherently subjective. For example, feeling more confident when holding a conversation or retaining the ability to cook a favourite meal is not comparable to an improvement in performance on a standardised cognitive test. The prescriber must translate the evidence base into perceivable benefits for the drug recipient. We identified that clinical experience, rather than research evidence, often provides the foundation for this – with implications for clinicians with less clinical experience to draw on. This also represents challenges for prescribing of future new dementia medications, where clinicians will lack clinical experience and where potential risks may be different.^22^

An important influencing factor as to whether a patient took dementia medication was how the risk and benefit information about drugs was presented, and for most participants the most influential source of information was the clinician. All respondents positioned medics as trusted experts, although as we have discussed, the way in which these experts managed questions and addressed concerns, or not, could leave uncertainties. Trust is documented to play an important role in the delivery of information about effectiveness of treatments.^23,24^ Trust was additionally important given the conscious (eg, *So .. yeah, I’m probably gearing it towards which way I prefer the patient to go, …*) and unconscious (eg, by way of emphasising or minimising side effects) steering observed. This finding highlights a potentially problematic tension between the patient’s role in the decision-making process, amplifying their autonomy beliefs and values as is best practice,^25^ and balancing this with the values, opinion and judgement of the clinician. These data suggest that the clinician’s judgment, although predicated on the best interest of the patient, has the potential to be at odds with shared decision making. For example, by taking a nuanced approached to the presentation of benefit or side effect information (*positioning* medication depending on the clinician’s belief about the best interest of the patients ie, steering), clinicians may limit the profile of information provided. That being said, the overall trust that patients articulated for clinicians can also be a regarded as a positive contribution to, and a valuable component of the shared decision making process.

Examples of *steering* observed in this study can be explained with reference to the concept of framing,^26^ where a decision is presented as having either a positive or negative outcome. This can apply to the communication of benefits or side effects. In this study there was evidence of the clinicians presenting the trials’ data in a positive frame indicating symptom improvement or delay of progression. There is evidence that the use of positive frame when presenting outcomes of ‘risky’ decisions can be more affirming for patients, lead to more positive perceptions of effectiveness^27^ and be more persuasive,^28,29^ To ensure communications about the effectiveness of treatments are informative, rather than persuasive it is essential for clinicians to have awareness of the possibility to influence using framing^30^

Positioning was seen to be especially influential concerning the presentation of side effects, which was a significant factor for both the clinicians and the patients/families. In general, it is acknowledged that the side effect profile is relatively mild, so even for people who do not see substantial benefits, on balance treatment is worth it. For many or most people the side effects are mild or self limiting so should not present as a barrier to prescribing. However, our findings did point to the importance of explicit consideration of risk, particularly for those who were frail and had comorbidities. One of our novel findings was that for some patients although the side effects might be mild and self limiting, for some people their degree of cognitive impairment alongside other health problems and frailty meant even minimal side effects such as gastro-intestinal issues, dizziness etc might impact an already fragile quality of life (for example by causing incontinence, increasing falls risk and hospitalisation) and ability to manage independently in day-to-day life – to the degree that even “trying it” was not perceived as worth the risk. In these cases, the perception was that potential side-effects of this type may disrupt the person’s routine and day-to-day life and the delicate balance of independence that they, their family or the doctor were concerned might not be recoverable. In these cases the risk of disturbing a delicate balance of independence maybe greater than the modest benefit of the drugs. This balancing of benefits against risks is likely to be even more complex for anti-amyloid treatment wherein the symptomatic benefits may not be substantially greater than the existing treatments.^22^ Although the side effect profile may involve greater risk and is a significant driver of the decision-making process for both clinicians and patients in our findings. Recent research corroborates these concerns suggesting a quarter of the general public (in their sample) expressed aversion to anti-amyloid treatments, with 70% of healthcare professionals citing concerns about brain bleeds and efficacy.^31^ That being said, these treatments are effective in modifying the disease pathology (namely amyloid clearance), and are targeted only to people in an early disease phase, who will not be experiencing all of the complexities associated with risk that are evident in our study population. Should these treatments be offered more widely exploring the side-effects and addressing concerns and understanding of these may be a core feature of the shared decision making process.

For those patients and supporters who recognised the benefits of the dementia medications were likely to be modest, and uncertain to be manifest, in the face of having no other alternative treatments for dementia, these drugs gave some hope and a sense of agency where otherwise they felt there was none; leading to the “give it a go” attitude observed. This hope and agency may also explain some of the relatively large (18%) placebo effect on cognition when prescribing AChIEs.^21^

Our findings also suggested that supporting information could be helpful or a hindrance in weighing up the risk and benefits. Written information was felt to be helpful in assisting decision-making, and for providing a lasting record of important information related to the medicine. Existing literature indicates patients value up to date, clear and concise information about the benefits of the treatments offered to them, preferably from a clinician, but leaflets and written information are also deemed helpful.^23,32^ Trust in the credibility of written information was important for prescriber and patient. However, not all sources of information online are reliable or accurate, and even reliable sources may be mis-interpreted. An example of this in our data is the case of Patient B003 who initially declined medication because of a fear they may experience side effects of aggression identified through an internet search on the drug. In the on-line British National Formulary provided by NICE, Aggression as is listed as a side effect of AChIEs, and as the side effects are reported alphabetically (Joint Formulary Committee 2023) and without data on incidence rates - it is given prominence in its presentation compared to other side effects, which may in fact be more frequently experienced and less concerning.

Owing to the legal obligation to present side effect information, generic official information leaflets within medication packaging tend to only present risks and side-effects; overlooking the presentation of benefits. This is likely to impact people with dementia more than other patient groups, as they may rely on written information about a drug in the face of memory problems. Having a better understanding of what information patients want and how to present it, as described in this paper will help inform clinicians and could improve medication uptake. This is especially pertinent as we face the potential future challenges of monoclonal antibody therapy; drugs which currently have fewer clear benefits (owing to an emerging evidence base) and more severe side effects.^22^ Conversely emerging medicines are set within a media context that position them as potentially game-changing drugs for treating Alzheimer’s disease. The use of relative risks ratio (ARR) and absolute risk ratios (ARR) can influence decision-making about treatments, with relative risks commonly presented in the media. This can lead to potential over estimations of benefit. Therefore, it is important that clinicians are prepared to respond to both patient over-expectations of the effectiveness of treatments and concerns about potential side-effects.

### Strengths and Limitations

This study was conducted within 2 NHS Trusts, during a time of ongoing and changing restrictions associated with the COVID-19 pandemic. Both NHS Trusts are based in North England, although in areas with differing socio-economic profiles. Clinicians self-selected participation in the study, therefore those who are less confident in discussing dementia medications may not be represented here. Furthermore, clinicians made initial contact with potential participants, and our sample may represent those for whom having conversations about dementia medications are more straightforward, or where there is likely to be better understanding of the aims of such medications. The COVID-19 pandemic is likely to have further magnified the difficulties people living with dementia face when accessing MAS, for example some services being delivered remotely and the difficulties of communicating when wearing facemasks. Our sample was predominantly White British, and all participants had at least 1 family member or friend supporting them during their appointment. The degree of trust in medics seen in this study may reflect the white-British demographics of participants. It is well documented that people from non-white British ethnic groups, have greater distrust of science and medical professionals due to experiences of racism and racial discrimination within healthcare^33^ and so their perspectives of clinical encounters and clinician influence on medication decision-making may differ. We also do not know how conversations may need to be adapted for those who do not have others supporting them. Further research should explore this, particularly for those who face additional barriers such as low health literacy or socioeconomic deprivation, which is associated with a poor-quality dementia diagnosis experience.

Another potential limitation is an observer effect owing to the data collection method – ethnography. To mitigate this, we engaged in extensive consultation about this as part of the ethics review process, took time to build relationships with staff and explain the purpose of the study, and used our extensive experience of ethnography to help reassure participants about the use of observations. Whilst there may have been some influence felt from being observed, observation of routine practice is common in clinical practice and training so this isn’t unusual, and the focus required to hold or participate in a clinical consultation tends to mean people often revert to their usual behaviour. Finally, owing to COVID-19 much of the data was collected using teams/audio only, so there was no researcher observer in the same room and the clinicians controlled the recordings, so this may by accident (rather than design) have helped to further mitigate any observer effects.

## Conclusions

Our findings point to a clear recommendation that clinicians need to be aware of their influence on the decision-making process, taking care to present balanced information that will promote shared decision making such as a clearly expressed articulation of their opinion based on clinical experience, alongside the opportunity for patients and their families to express their beliefs and qualify their understanding of treatments offered. That said, our findings did show some encouraging signs of shared decision making – such as trust in the clinician and patients and relatives articulating their treatment preferences clearly. Colleagues with less experience might be provided with shared clinical expertise to draw on alongside facts and figures from research.

### Recommendations for Practice

This study has contributed several recommendations for practice described in Table 2. Prescribers in memory clinic would benefit from a shared standardised information source that clearly defines the benefits and risks of available medications. It should be designed with service users and their carers, updated regularly and available in multiple languages and formats to account for sensory deficits. The information should be evidence based and endorsed by national bodies, for example the memory services national accreditation programme (MSNAP).

**Table 2.** Overview of Recommendations.

Recommendations	Suggestions for Implementation
To make transparent and accessible information about side effects AND benefits available to both clinicians and patients, and effectively communicate this to patients	
Provide standardised information for patients and carers that clearly defines the benefits and risks of available medications	- Ensure communications are accessible to people with low health literacy – following recommendations such as the universal precautions toolkit^34^ - Give information in short simple bullet points - Include visuals to explain likelihood of benefits and risk - Update regularly and make available in multiple languages and formats - Make information available through third sector support organisations (eg, Alzheimer’s society) as well as through healthcare pathways (such as sending with MAS appointment invitation)
Provide standardised information for clinicians that clearly defines the benefits and risks of available medications with reference to clinical trial data	- Ensure information is up to date with reference to latest evidence - Provide visuals to explain likelihood of benefits and risk - Obtain endorsement from national bodies
Ensure that prescribers have access to training education on the understanding clinical trial outcomes and approaches to communicating likelihood of benefit of risk of harm to patients in a meaningful way	- Provide communication skills and critical appraisal skill training, as part of their continuous professional development - Provide colleagues with less experience with shared clinical expertise to draw on alongside facts and figures from research - Promote staff access to high-quality dementia training tailored to their role to increase staff knowledge and confidence
Promote shared decision making about dementia medications	
Provide time for patients and supporters to understand the uncertainties associated with dementia medication and make informed decisions	Allow for at least 1 follow up session after FIRST providing information about the medications which includes the opportunity to discuss the risks, benefits and uncertainties of treatments
Where patients/relatives have difficulty understanding medication information promote the use of advocates where appropriate	Assess the patients supporters understanding of information about medications and explore access to/use of advocates where needed/available
Awareness of positioning and influence of this	Provide standardised training and information to increase awareness of the influence of positioning on medication decisions and uptake
Use resources and techniques known to promote shared decision making	Make use of written resources such as patient decision aids Use “teach back” techniques to assess and check understanding, reasons for prescribing/not prescribing and potential side effects, how to manage them, how long the medication will be prescribed for/ how it can be deprescribed

The majority, but not all prescribers will have had medical and psychiatric training. The Royal College of Psychiatrists places a large emphasis on the critical appraisal of research and communications skills in the curriculum. There should be continued drive for clinicians working in memory clinic to receive communication skills and critical appraisal skill training, as part of their continuous professional development. This should include education on the understanding clinical trial outcomes and approaches to communicating likelihood of benefit of risk of harm to patients in a meaningful way.^35^

For there to be a greater level of shared decision-making there is a need for more time explaining the complexities of the risks, benefits and uncertainties of treatments to patients. This could include, where necessary, the use of advocates to facilitate decision making.

## Consent for Publication

No personal individual data is presented. Participants consented to use of direct quotes from transcripts to be used in publication.

## Data Availability

The datasets used and/or analysed during the current study are available from the corresponding author on reasonable request.*
